# Pore timing: the evolutionary origins of the nucleus and nuclear pore complex

**DOI:** 10.12688/f1000research.16402.1

**Published:** 2019-04-03

**Authors:** Mark C. Field, Michael P. Rout

**Affiliations:** 1 School of Life Sciences, University of Dundee, Dundee, UK; 2 Biology Centre, Institute of Parasitology, Faculty of Sciences, University of South Bohemia, Ceske Budejovice, Czech Republic; 3 The Rockefeller University, New York, USA

**Keywords:** eukaryogenesis, vesicle coats, nuclear pore complex, molecular evolution

## Abstract

The name “eukaryote” is derived from Greek, meaning “true kernel”, and describes the domain of organisms whose cells have a nucleus. The nucleus is thus the defining feature of eukaryotes and distinguishes them from prokaryotes (Archaea and Bacteria), whose cells lack nuclei. Despite this, we discuss the intriguing possibility that organisms on the path from the first eukaryotic common ancestor to the last common ancestor of all eukaryotes did not possess a nucleus at all—at least not in a form we would recognize today—and that the nucleus in fact arrived relatively late in the evolution of eukaryotes. The clues to this alternative evolutionary path lie, most of all, in recent discoveries concerning the structure of the nuclear pore complex. We discuss the evidence for such a possibility and how this impacts our views of eukaryote origins and how eukaryotes have diversified subsequent to their last common ancestor.

## Introduction

The origin of eukaryotes occurred over one and a half billion years ago. Unlike prokaryotes (Archaea and Bacteria), eukaryotic cells possess a complex and differentiated endomembrane system: the endoplasmic reticulum (ER), the Golgi complex, vacuoles, and families of coating complexes to form vesicles that traffic between them. Recent findings have allowed us to reconstruct the evolutionary history and development of these endomembrane systems from their ancient prokaryotic ancestors. Protein families that are central to the construction and function of endomembrane compartments include Rab and ARF (ADP-ribosylation factor) small GTPases; vesicle coat proteins such as clathrin, COPI, and COPII; and the ESCRT (endosomal sorting complexes required for transport) proteins ^1– 5^. Crucially, with the exception of ESCRT (which appears to have undergone minimal expansion despite acquiring multiple roles) ^6^, these eukaryotic families arose through paralogous expansions from their ancestral prokaryotic genes ^1, 3^.

**Figure 1.**
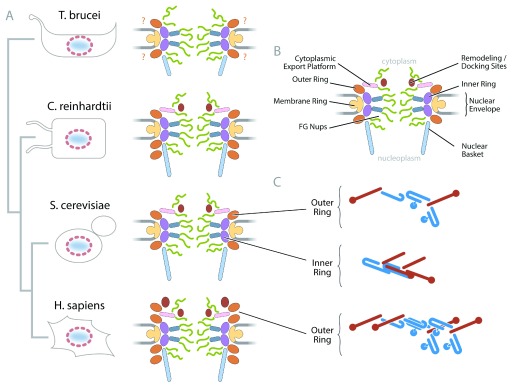
Diversity within modern nuclear pore complex (NPC) structures. ( **A**) Four NPC examples are shown from different taxa, metazoa, fungi, green alga, and kinetoplastids (some elements speculative in the latter), and approximate evolutionary relationships are shown at the far left. ( **B**) A schematic of a generic NPC, based on the *Saccharomyces cerevisiae* structure, is shown at the top right. ( **C**) Variations in the scaffold structure, export platform, cytoplasmic face remodeling complex, and the nuclear basket structures are indicated. The outer and inner ring nucleoporin (Nup) complex subunit arrangements are shown at the bottom right, emphasizing the distinct coat architectures present. Type I coats are red and type II are blue. FG, phenylalanine-glycine.

Of course, the largest and indeed the defining endomembrane compartment of eukaryotes is their nucleus, which contains and organizes almost all of the cell’s genetic material. The modern eukaryotic nucleus is bounded by a double-membrane nuclear envelope (NE), which is contiguous with, and functionally related to, the ER. Embedded in the NE are nuclear pore complexes (NPCs), which are among the most recognizable and defining macromolecular assemblies associated with the nucleus. The NPCs of all eukaryotes studied so far share a basic bauplan—a minimal set of common architectural features—although there are significant lineage-specific variations ^7– 10^ ( Figure 1). Each NPC is composed of about 30 different kinds of proteins, termed nucleoporins (or Nups), present in about 500 copies that form an annular structure of between 50 and 100 MDa (depending on the organism) with an overall eight-fold radial symmetry ^7, 8, 10– 14^. At the heart of the NPC is a core scaffold composed of coaxial outer and inner rings that in turn are connected to a membrane ring. Having a nuclear basket and an export platform at the cytoplasmic face, the NPC is asymmetric along its cylindrical axis. Collectively, all these discrete, relatively rigid assemblies are linked by flexible connector cables, an architecture that imbues the NPC with both flexibility and strength ^10, 14– 18^. The scaffold surrounds a central channel, whose inner wall is lined with nucleoporins termed FG (phenylalanine-glycine) Nups, from which project domains with multiple, intrinsically disordered FG repeat motifs that fill the channel and form a region termed the central transporter. These FG repeats mediate selective nucleocytoplasmic transport through specific interactions with nuclear transport factors, which carry their cognate macromolecular cargoes ^14, 19– 21^.

It remains unclear whether the nucleus arose as the first intracellular organelle or whether other organelles were already present within transitional eukaryotes (that is, those prior to the emergence of the last eukaryotic common ancestor, or LECA). The complexity of the modern “true” nucleus does suggest that the current form was unlikely to have been the ancestral state or present at the beginning of eukaryogenesis. We can safely assume that early nuclear forms were simpler structures but that they provided some selective advantage (see below). Eukaryotes also possess mitochondria, which originally were bacterial and were gained through an endosymbiotic event. However, the point at which mitochondrial endosymbiosis occurred relative to nuclear origin has been unclear. For example, while robust arguments for early mitochondrial origins, to provide sufficient energy for synthesis and maintenance of the sophisticated eukaryotic endomembrane system, have been made ^22, 23^, others have countered that a phagocytic apparatus is essential for the mitochondrial progenitor to have invaded the host cell ^24– 26^ and that phylogenetic evidence also suggests a late mitochondrion ^27^.

Critically, evolution of the endomembrane system and integration of the mitochondrial bacterial ancestor with the host cell are events requiring a great deal of time but are not necessarily coupled, such that the two processes could actually occur in parallel or in series. A focus on the timeline of mitochondrial origins also overlooks another origin question—that of the nucleus. Reasonably, it has long been assumed that the nucleus, being the defining organelle of eukaryotes, arose very early around the time of the first eukaryotic common ancestor (FECA) and elaborated to the structure we recognize now by the time that LECA arose ^28^. But is this actually the case? Recent work on the architecture of vesicle coating complexes and the NPC challenges this assumption and suggests alternate possibilities.

## Origins of the first membrane coating complex

**Figure 2.**
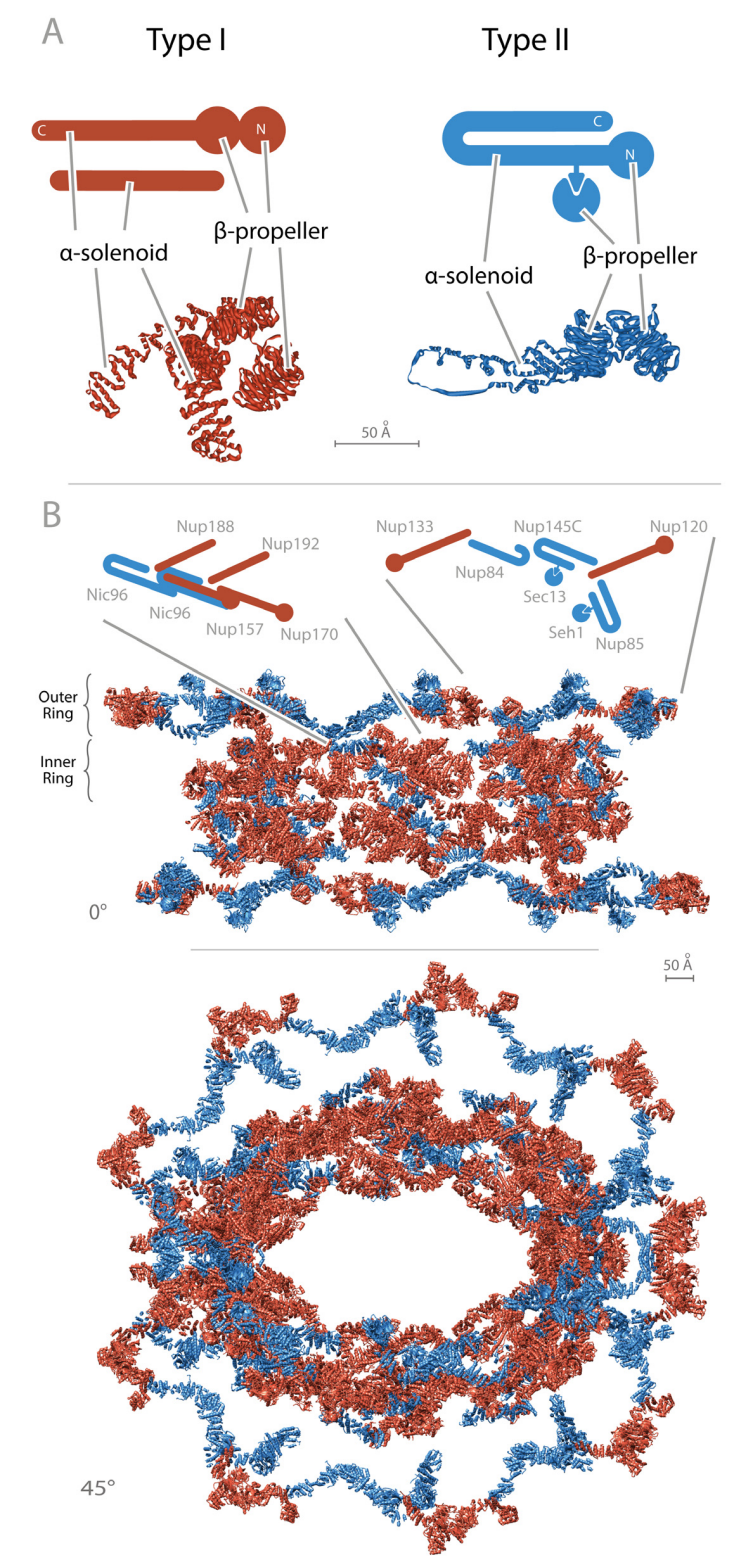
Type I and type II coat protein architectures and locations within the nuclear pore complex. ( **A**) Examples of type I and type II architectures are shown. Type I coats are red and type II are blue. Idealized structures appear above, and examples of structures determined by x-ray (COPI subunits pdf:5A1U and COPII subunits pdf:4BZJ as representatives of type I and type II, respectively) appear below. Note the characteristic β-propeller head and α-solenoid tail in both type I subunits, the α-solenoid adaptin-like subunit in type I, and the clear presence of a discontinuity and loop in the α-solenoid following the β-propellers in type II. ( **B**) A section of the *Saccharomyces cerevisiae* nuclear pore complex structure is shown at the bottom, illustrating only the type I– and type II–related subunits. Note the intermixing of types within the overall structure.

Trafficking between the endomembrane systems is mediated by complexes that coat membranes to induce budding of vesicles as well as to define and stabilize the compartments themselves. The great majority of these coating complexes contain α-solenoid and β-propeller folds and the characteristic combination of an N-terminal β-propeller and C-terminal α-solenoid (that is, β-α) ( Figure 2). Furthermore, all coating complexes are associated with, and regulated by, small GTPases, albeit with diverse mechanisms (reviewed in 3). The ubiquity of these features indicates that all coating complexes in this extensive group must have originated from a common ancestor, early in the path from FECA to LECA and with progenitors pre-FECA. Some evidence of this may have been found in the Asgard archaea lineage; members of this lineage are suggested to possess genes encoding proteins similar to the B-propeller fold protein Sec13 and α-solenoid-containing proteins as well as Rab-like GTPases, the basic building blocks required for a primitive vesicle coat ^1, 29, 30^.

## Elaboration of the coating and endomembrane systems

At least two major families of the β-α class of vesicle coating protein can be differentiated, each having discrete vesicle recognition and trafficking roles. The type I family contains the COPI coating complex, which largely mediates Golgi-to-ER trafficking together with various complexes based around clathrin, which mediate trafficking from the plasma membrane to endosomes and additional endosomal pathways. The type II family contains the COPII complex, which mediates ER-to-Golgi trafficking ^4, 31^. Phylogenetic reconstructions for the type I family exhibit a clear path of expansion, which likely reflects the evolution of increasingly complex post-Golgi trafficking systems during the period between FECA and LECA ^4^.

Each family carries a distinct arrangement of the β-α folds. The type I family has coatomers with a (more or less) straight α-solenoid following the β-propeller fold region; several associated adaptins responsible for protein sorting within the endomembrane system also have an α-solenoid fold. The type II family has coatomers possessing α-solenoids with a characteristic U-turn topology, following the β-propeller fold region and also associated smaller proteins containing only a β-propeller fold ( Figure 1 and Figure 2) ^5, 31– 36^. These distinct and conserved morphologies of the type I and type II families indicate that the next step after development of a vesicle coating system was divergence into two coating families with distinct architectures and functions in what therefore must have been by this point a differentiated membrane system (that is, one in which distinct compartments—presumably progenitors of the ER, Golgi, and possibly endosomes—were present) ^3, 4^.

## Evolutionary origins of the nuclear pore complex

Recent high-precision structures from different species have allowed a much more detailed look at how the NPC might have originated ^7, 8, 10, 13^. As well as forming the structural foundation of the NPC, the core scaffold serves to coat and define the shape of the pore membrane, the membranous fenestration in the NE within which the NPC sits. The NPC’s resemblance to a vesicle coat does not end there; indeed, essentially, the entire core scaffold is constructed from Nups that share their folds with those of the vesicle coating complexes, and the NPC even contains one protein shared with the COPII coat (Sec13) and another (Seh1) shared with the coat-related SEA (Seh1-associated) tethering complex. This clear relationship between NPCs and vesicle coating proteins led to the “protocoatomer” hypothesis, proposing that a huge number of different membrane-associated complexes have a common evolutionary origin. Originally, this focused on the NPC and the COPI, COPII, and clathrin complexes that coat and stabilize curved membranes but recently has been extended to include several tethering complexes and the intraflagellar transport system ^37– 40^. Nevertheless, all of these complexes still carry proteins with only either type I or type II fold arrangements.

Strikingly, though, the NPC stands out from all other known coat complexes as containing proteins with both type I and type II features ( Figure 1 and Figure 2). There are two possible routes by which this could have arisen; specifically, a progenitor NPC was formed from exclusively type I or type II subunits and then acquired additional subunits of the other type, or the NPC was a mélange of type I and type II from the start. The first possibility would indicate the evolution of a primitive NPC quite early in eukaryogenesis and possibly even prior to the split between the type I and II architectures, but the second model emphatically requires a later origin after the establishment of the two coat types.

**Figure 3.**
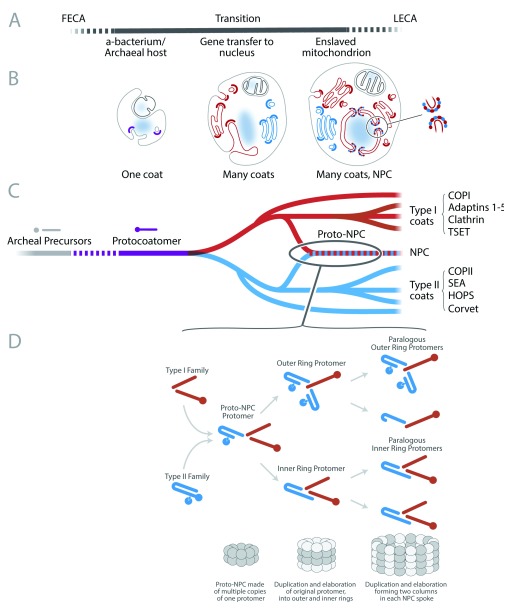
Timeline for evolution of coat complexes, mitochondrial enslavement, and nuclear pore complex (NPC) elaboration. ( **A**) The top system illustrates acquisition and gradual enslavement of the mitochondrion by transfer of genes from the original bacterial genome to the genome of the host cell. Dotted lines indicate uncertainty concerning both the point of endosymbiont acquisition and duration of the overall process. ( **B**) The top middle system depicts hypothetical general structures of transitional eukaryotic cellular forms at various stages during the process of eukaryogenesis. The location and type of protocoatomer are indicated by colored crescents. Note the amalgam coat of the NPC. ( **C**) The lower middle system is a proposed timeline for the evolution of vesicular coat complexes, colored to correspond with the cellular diagrams. The earliest proto-eukaryote is suggested to have a single ancestral coat (protocoatomer), which is derived from Archaeal genes. In many prokaryotes, such genes are present, but the β-propellers and α-solenoids are not fused to encode a single protein. Examples of type I and type II architectures are shown at the right, together with examples of specific complexes present in modern eukaryotes. ( **D**) The lower system illustrates a possible route for the evolution of the NPC, placing emphasis on the duplication of subunits and merging of protocoatomer type I and type II to create the modern NPC structure. Ovals represent subunits in the evolving NPC and are differently shaded to indicate duplications. Type I coats are red, type II are blue, and protocoatomer is purple. Putative archaeal lineages and proteins are shown in gray. FECA, first eukaryotic common ancestor; LECA, last eukaryotic common ancestor.

Is there evidence that favors one of these two models over the other? Clues that collectively discriminate between these two models can again be found in the scaffold architecture of the NPC, which contains remnants or “molecular fossils” of its precursors. There is a repetitive modularity within the core scaffold. It is made of eight spokes, each of which can be divided vertically into two paralogous columns that clearly arose through duplication. The inner and outer rings are also structurally related. Although the order of these duplications cannot be fully resolved, it is clear that an early NPC contained a subunit whose early duplication and divergence event presumably led to paralogous subunits ( Figure 3) ^8, 10, 11, 41^. This is because the spokes, columns, and inner and outer rings share similar architectures and folds. Thus, the inner and outer rings each contain truncated versions of a COPI-like coatomer, a COPII-like coatomer, and a coiled-coil bundle. For example, these duplications can be seen in the recently solved yeast ( *Saccharomyces*) NPC structure ( Figure 2). The inner ring Nic96 subunit and outer ring Nup84, Nup85, and Nup145C subunits have the distinctive solenoid architecture of type II coatomer subunits found in Sec31 ^42^. Indeed, Nup145C associates with Sec13, itself a bona fide COPII subunit. In contrast, the inner ring Nup170 and Nup157 subunits together with the outer ring Nup120 and Nup133 components all have characteristic type I architectures. Moreover, Nup170 and Nup157 associate with two adaptin-like Nups, respectively Nup192 and Nup188, a subcomplex that bears similarities to the clathrin coat system. The major family of soluble cargo-carrying nuclear transport factors collectively termed karyopherins share this adaptin-like structure, suggesting that they also originated from, and co-evolved with, the elaborating NPC ^38, 43, 44^.

## A late evolutionary origin for the nucleus

Crucially, all of these paralogous subunits now scattered throughout the NPC scaffold retain signatures of both type I and type II families, which implies that the original progenitor NPC formed through an amalgamation of both type I and II families of coating complex. This in turn implies that ancestral type I and type II coating families evolved first, together with an already-differentiated internal membrane system, before the modern NPC had arisen. However, these structures would be lacking any of the key defining features and functions that typify NPCs and NEs in extant organisms. The model implies that a true nucleus, which is structurally and compositionally defined largely by the NE and embedded NPCs, was a later addition on the path to the evolution of early eukaryotes. This is a radical departure from most previous eukaryogenesis models, which have focused on the relative timing of the acquisition of the mitochondrion versus the endomembrane system. Most significantly, this new model also considers a more gradual progression for both nuclear and mitochondrial origins prior to the eventual emergence of the modern form of the nucleus ^28^. FECA, and many intermediates between it and LECA, will not have possessed a modern nucleus or many of the other features considered characteristic of eukaryotes; rather, these features were “works in progress” until comparatively late in eukaryogenesis.

## Diversification of the nuclear pore complex

In just the last few years, there has been an explosion of information concerning the composition, stoichiometry, and even the fine structure of NPCs from divergent taxa, allowing a more detailed view of the likely LECA NPC structure and how this can diverge in response to the distinct lifestyles of different organisms. Protein components of the NPCs from various species have been determined at varying levels of completeness and include *Schizosaccharomyces pombe* ^45^, *Chaetomium thermophilum* ^15^, *Arabidopsis thaliana* ^46^, *Tetrahymena thermophila* ^47^, *Trypanosoma brucei* ^48^, *Homo sapiens*, and *Rattus rattus* ^49, 50^, and more detailed EM analyses are available for *Saccharomyces cerevisiae*, *Homo sapiens*, *Xenopus laevis*, and *Chlamydomonas reinhardtii* ^7, 8, 10, 51^.

Originally, it was thought that there had been considerable conservation of NPC architecture despite extensive sequence divergence of individual Nups ^48^. However, this view now seems premature, and new analyses of NPCs from multiple species paint a more complex picture ( Figure 1 and Figure 3) ^7– 10, 13^. Rather, a “one size fits all” model ^17^ is clearly inaccurate and, even while there is indeed an underlying and conserved bauplan, the NPC is composed of modules (each containing just a few Nups) which can be assembled in a surprisingly large variety of arrangements and specializations, likely often through the same processes of duplication and divergence that led from the post-FECA proto-NPC to the full LECA NPC. This is highly reminiscent of LEGO™, where many different forms can be easily created from the same small set of building blocks. Significantly, NPCs even have distinct compositions in different tissues, attesting to the careful tuning of NPC structure to function in a specific cellular context ^52– 54^.

A comparison of a recently determined vertebrate scaffold structure with the scaffold from the complete yeast NPC structure illustrates these processes ( Figure 1) ^8, 10^. There are yeast paralogs (for example, Nup170 and Nup157) absent from vertebrates (only Nup155). Vertebrates also carry duplicates of the entire outer ring such that there are 32 copies of the complex that comprises this ring and so there are four outer rings per NPC in vertebrates while there are 16 such complexes and two outer rings in yeast. Vertebrates also have extra copies of the inner ring Nup155 and possibly Nup188 or Nup205 (Nup157/Nup170, Nup188, and Nup192, respectively, in yeast). These and other vertebrate-specific additions and alterations mean that, at about 109 MDa, the vertebrate NPC likely weighs in at around twice the 52.3-MDa yeast NPC. However, we find that the total mass of the central transporter FG repeats is surprisingly constant: about 9 MDa in yeast, human, and trypanosome NPCs (although this is based on assumptions in terms of stoichiometry of the trypanosomal FG-Nups) ^8– 10^. Even the proportion and distribution of certain types of FG repeat seem conserved,—there being mainly either “FXFG” (phenylalanine-X-phenylalanine-glycine) or “GLFG” (glycine-leucine-phenylalanine-glycine) types of repeats. It seems possible that the central transporter—the core machinery of transport—has strict bounds in terms of mass, density, and composition even if the precise sequence and arrangement of FG repeats may vary between species.

Drastic alterations of the outer ring subcomplex are also found in other organisms, indicating that this module of the NPC is particularly susceptible to lineage-specific modulation ( Figure 1). Perhaps this is because the outer ring, being involved mainly in NE attachment and shaping the NPC and having little direct involvement in defining the central transporter machineries, is less structurally and functionally constrained ^10^. Unlike in *S. cerevisiae* and vertebrates, in another yeast, *S. pombe*, the relative stoichiometry of outer ring Nups is non-uniform between the cytoplasmic and nuclear facing subcomplexes, indicating a significant deviation in outer ring complex architecture ^45^. Further afield, NPCs in the micronuclei of the ciliate *T. thermophila* differ significantly in composition from those in macronuclei, and numerous Nup paralogs are specifically localized to one or the other type of nucleus ^47^. In the algae *C. reinhardtii*, cryogenic electron microscopy (cryo-EM) revealed that there are two outer rings on the NPC nuclear side but only one on the cytoplasmic side. Moreover, the diameter of the inner ring and central channel is significantly larger than in either yeast or vertebrates ^7^. There is also considerable variation in the *trans*-membrane protein identities between these organisms.

Finally, in the highly divergent trypanosomes, there are a number of Nups with no obvious homologs in other taxa. The trypanosome NPC also lacks *trans*-membrane Nups with any discernable evolutionary relationship to membrane Nups in other organisms and the entire NPC arrangement contrasts with those of other organisms in being almost entirely symmetric in the deployment of the FG repeat-containing Nups around a cylindrical axis ^9^. Even greater variation is found in the more peripheral NPC structures, the nuclear basket, and cytoplasmic export platform. The size of the core coiled-coil basket-associated proteins varies greatly between species, ranging from about 200 kDa in Opisthokonta to only about 100 kDa in trypanosomes ^55^. In the cytoplasmic export platform, there is huge variation in the type and number of docking sites for accessory transport proteins. Trypanosomes have a simple peripheral organization and lack any obvious transport factor docking sites beyond the FG repeats ^9^. In *S. cerevisiae*, docking sites are present on the NPC cytoplasmic side for several RNA remodeling proteins, including Gle1 and Dbp5; the lack of both of these docking sites and genes encoding the remodeling proteins in trypanosomes is likely a reflection of distinct mRNA splicing mechanisms, as previously discussed ^3, 56^. In vertebrates, elaborate arrays of docking sites exist on the cytoplasmic platform, both for such RNA remodeling proteins and for nuclear transport accessory proteins such as Ran, RanGAP (Ran GTPase-activating protein), and Ubc9 (ubiquitin-conjugating gene product 9) ^57, 58^. All of these examples of dramatic lineage-specific NPC structures underscore the need for direct, high-resolution structural and functional studies in multiple organisms, which are based on a firm evolutionary foundation, rather than drawing conclusions on the demonstrably false premise of structural interchangeability between divergent species; the latter is the equivalent of taking cod bones, stuffing them in a puffin skin, and calling it an accurate model of Leifur Eiriksson.

## Implications

The division between prokaryote and eukaryote cellular architectures represents a great divide in the evolution of life on earth, and the process of eukaryogenesis is recognized as one of life’s major evolutionary transitions ^59^. Many innovations were required to achieve this, including the formation of an elaborate and differentiated endomembrane system, a complex cytoskeleton, endosymbiosis and integration of the mitochondrion, and the formation of a nucleus with a nuclear transport system to ferry cargoes across the NE via the NPC. The relative order of emergence of these innovations has remained both an important aspect of understanding eukaryogenesis and an area of intense debate. Most models tend to gravitate around two alternative scenarios. In the first, it is suggested that there was a prior need for a mitochondrion in order to power the evolution of subsequent cellular innovations; in the second, it is countered that this power source was not needed and that the cell was already well on the way toward an elaborate eukaryotic cellular architecture before it gained a mitochondrion ^22– 26^. However, we suggest an alternative third model, both plausible and supported by recent data from NPC structures, where both mitochondrial enslavement and elaboration of an endomembrane system occurred roughly in parallel during the transition from the Archaea to LECA ( Figure 3). This process started with a single “protocoatomer” to begin the formation of endomembranes, which duplicated and diverged into the ancestors of the type I and type II families of coating complexes in order to help drive the diversification of the endomembrane system into the ancestors of the Golgi, the ER, and endosomes. Members of the type I and type II families came together in a partnership to form the first proto-NPCs in a differentiating NE. Our model implies that the nucleus, the organelle defining the name “eukaryote” (“with nucleus”), was actually a significantly later addition on the path to the evolution of the early eukaryotes, after the formation of differentiated endomembranes and coating systems. This should also be placed in the context of other events that could have taken place concurrently (for example, transfer of genes from the mitochondrial endosymbiont to the nucleus and the formation of the cytoskeleton). In a very real sense, this questions the concept of FECA as a fixed point on the pathway of eukaryogenesis.

What would the state of a nucleus have been in the transitional period, and why did the NPC arrive at the present architecture? We have previously argued that a proto-NPC could have acted as a membrane organizer such that the genome became associated with a rather open membrane—containing fenestrations stabilized by a primitive assembly—that at some point recruited both type I and type II coating complex family members ^40^. This structure would have provided some mechanical protection for the genome as well as the potential beginnings of organization at the NE. Such a system had to be retrofitted to accommodate the selective transport of thousands of macromolecules that previously enjoyed free access to the genome. The evolutionary origin of the selective permeability barrier may also have been a relatively simple modification of existing elements: the flexible connectors could have expanded to form the FG repeat regions (many of which are still associated with flexible connector regions), and the α-solenoid adaptin-like Nups that the connector cables attach to could have evolved to form the karyopherin family of transport factors, which they still closely resemble ^38, 43, 44^. It is unknown whether the karyopherins evolved early pre-LECA, although the diverse families indeed were present at that time ^60^. In summary, the NPC is a prime example of the evolution of evolvability, superbly adaptable by specific lineages. These changes are clear from the ancient history of the NPC as inferred for the origins of the structure present in the LECA as well as continuing to the present day, with variation of NPC structures in distinct lineages or tissues now being widely reported.

## Abbreviations

COP, coatomer protein; ER, endoplasmic reticulum; ESCRT, endosomal sorting complexes required for transport; FECA, first eukaryotic common ancestor; FG, phenylalanine-glycine; LECA, last eukaryotic common ancestor; NE, nuclear envelope; NPC, nuclear pore complex; Nup, nucleoporin
